# A prospective randomised controlled mixed-methods pilot study of home monitoring in adults with cystic fibrosis

**DOI:** 10.1177/17534666211070133

**Published:** 2022-03-11

**Authors:** Edward F. Nash, Jocelyn Choyce, Victoria Carrolan, Edwin Justice, Karen L. Shaw, Alice Sitch, Hema Mistry, Joanna L. Whitehouse

**Affiliations:** West Midlands Adult CF Centre, University Hospitals Birmingham NHS Foundation Trust, Bordesley Green East, Birmingham B9 5SS, UK; West Midlands Adult CF Centre, University Hospitals Birmingham NHS Foundation Trust, Birmingham, UK; West Midlands Adult CF Centre, University Hospitals Birmingham NHS Foundation Trust, Birmingham, UK; West Midlands Adult CF Centre, University Hospitals Birmingham NHS Foundation Trust, Birmingham, UK; Institute of Applied Health Research, College of Medical and Dental Sciences, University of Birmingham, Birmingham, UK; Institute of Applied Health Research, College of Medical and Dental Sciences, University of Birmingham, Birmingham, UK; NIHR Birmingham Biomedical Research Centre, University Hospitals Birmingham NHS Foundation Trust and University of Birmingham, Birmingham, UK; Clinical Trials Unit, Warwick Medical School, University of Warwick, Coventry, UK; West Midlands Adult CF Centre, University Hospitals Birmingham NHS Foundation Trust, Birmingham, UK

**Keywords:** cost-effectiveness, cystic fibrosis, health-related quality of life, HM, pulmonary exacerbations

## Abstract

**Background:**

Home monitoring (HM) is able to detect more pulmonary exacerbations (PEx) than routine care (RC) in individuals with cystic fibrosis (CF), but there is currently no evidence for benefits in health outcomes. Patient experiences of using HM and a health economics assessment have not been rigorously assessed to date. This study aimed to assess the effects of HM on hospital admissions, quality of life, antibiotic requirements, exacerbation frequency, lung function, nutritional outcomes, anxiety, depression, costs and health outcomes, as well as the qualitative effects on the patient experience.

**Methods:**

This randomised controlled mixed-methods pilot study recruited CF adults cared for in one large regional CF centre. Participants were randomly allocated 1:1 to the intervention cohort [twice-weekly HM of symptoms measured by the Cystic Fibrosis Respiratory Symptom Diary – Chronic Respiratory Infection Symptom Score (CFRSD-CRISS) and forced expiratory volume in one second (FEV_1_)] or a control cohort (routine clinical care) for the 12-month study period. Measurements were recorded at study visits at baseline, 3, 6, 9 and 12 months. Spirometry, body weight, comorbidities, medications, hospital inpatient days, courses of antibiotics (oral and intravenous) and PEx (defined by the modified Fuchs criteria) were recorded at each study visit. Health status, capability and cost-effectiveness were measured at each study visit by the Hospital Anxiety and Depression Scale (HADS), the ICEpop CAPability measure for Adults (ICECAP-A), EuroQol 5 dimensions (EQ-5D-5L) questionnaire and an adapted resource use questionnaire. The patient experience of HM was assessed by semi-structured qualitative interviews at baseline and 12 months.

**Results:**

Eighty-eight participants were recruited, with 44 (50%) randomised to receive HM and 44 (50%) randomised to receive RC. Patient hospital inpatient bed days per annum and overall health-related quality of life were similar between the groups. Protocol-defined PEx requiring intravenous and oral antibiotics were detected more frequently in the HM group, with no other differences between the groups in the secondary outcomes. The total mean National Health Service (NHS) costs were approximately £1500 more per patient for the RC arm than the HM group. The qualitative analysis demonstrated that the patient experience of HM was generally positive and overall the intervention was well accepted.

**Conclusion:**

The findings of this trial confirm that HM is effective in detecting PEx in adults with CF. There were no significant differences in hospital inpatient bed days and overall health-related quality of life between the groups. Despite the cost of the HM equipment and the salary of the research fellow to respond to the results, health economics analysis suggests the intervention was less expensive than RC. HM was generally well accepted, with most participants reporting that it resulted in them feeling more empowered and reassured.

**Trial registration:**

The study protocol was registered with Clinicaltrials.gov (NCT02994706) on 16 July 2014 and published in a peer reviewed journal.

Data from this trial has been presented in abstract form at the ECFS Conference in Lyon in September 2020.

## Background

People with cystic fibrosis (CF) typically suffer a progressive decline in lung function, resulting in premature mortality, most commonly due to respiratory failure.^1^ Intermittent episodes of acute worsening of symptoms, termed ‘pulmonary exacerbations’ (PEx), are common and result in impaired quality of life.^2^ Patients with more frequent PEx experience a more rapid decline in lung function and have a worse prognosis.^3^ In 25% of PEx, the patient suffers a permanent and irrecoverable loss of lung function.^4^ Factors predictive of failure to return to baseline include being female, pancreatic insufficiency, *Pseudomonas aeruginosa*/*Burkholderia cepacia* complex/methicillin-resistant *Staphylococcus aureus* (MRSA) infection, being undernourished, allergic bronchopulmonary aspergillosis (ABPA) and larger drop in forced expiratory volume in one second (FEV_1_) before treatment initiation.^4^ The majority of these factors cannot be prevented, but theoretically if exacerbations could be diagnosed earlier, before patients have lost weight and FEV_1_, it may be possible to reduce or even prevent permanent loss of lung function. Since symptoms often gradually deteriorate during a PEx,^5^ there has been interest in using home monitoring (HM) to start treatment more promptly and to potentially improve the outcomes of people with CF.^6–17^

The recent Early Intervention in Cystic Fibrosis Exacerbation (eICE) randomised controlled trial confirmed that HM was able to detect more PEx than routine care (RC), with PEx detected by HM being more likely to be treated with oral antibiotics and less likely to require intravenous antibiotics.^17^ However, this study found no evidence of slowing in lung function decline over the 12-month study period. Despite this finding, interest remains in evaluating the potential for HM to improve health outcomes, particularly given the growing number of people with CF and challenges with providing regular clinical evaluation of this patient group. The health economics impact and the patient experience of receiving HM have also not been thoroughly assessed in previous research.

The primary aims of this study were to (1) determine whether HM is effective compared with RC in reducing hospital inpatient bed days and (2) assess whether this results in differences in health-related quality of life (HRQoL) over a 12-month period in adults with CF. We hypothesised that participants randomly allocated to HM would require fewer hospital inpatient bed days and that they would have better HRQoL compared with those receiving RC.

## Methods/design

### Study design

This single-centre, nonblinded, randomised controlled mixed-methods trial was conducted at West Midlands Adult CF Centre, University Hospitals Birmingham National Health Service (NHS) Foundation Trust, Birmingham, UK.

### Participants

#### Inclusion/exclusion criteria

To be eligible for enrolment, participants had to be able to give informed consent, be aged 18 years or older, have a diagnosis of CF confirmed by clinical characteristics, sweat test and/or genetic testing, be clinically stable at the time of recruitment (as assessed by the treating physician) and have a history of at least one admission to hospital to receive intravenous antibiotics over the preceding 24 months.

Exclusion criteria include (1) currently participating in another clinical trial (excluding observational studies); (2) pneumothorax or lung surgery within the previous 3 months or eye surgery (e.g. cataract operation) in the previous 4 weeks (because these factors prevent measurement of spirometry); (3) airway infection with *Burkholderia cenocepacia* or *Mycobacterium abscessus* at the time of recruitment; and (4) previous lung transplantation procedure.

#### Sample size

Estimating the mean number of hospital inpatient days is 42 for those receiving standard care and 36 for those receiving monitoring, Poisson exact 95% confidence intervals for incidence rate estimates would be (40.0, 44.1) and (34.2,37.9), respectively. To allow for patient drop out, we aimed to recruit 100 patients (50 patients in each cohort).

#### Recruitment and randomisation

**Figure 1.**
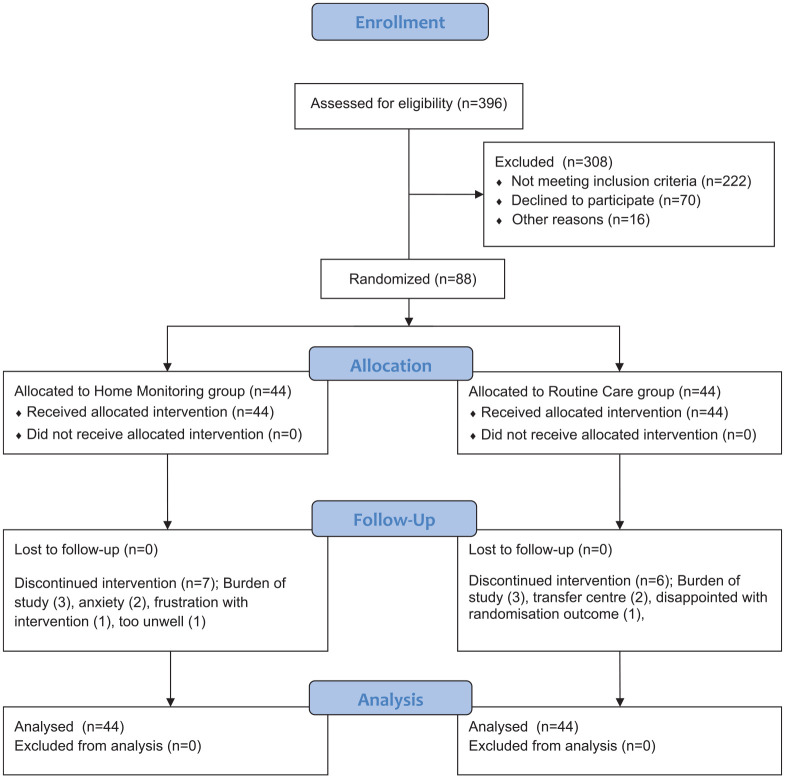
Flow of patients through the study.

The flow of participants through the study reflected the recommendations from the Consolidated Standards of Reporting Trials statement^18^ as outlined in Figure 1. Participants received written and verbal information explaining the study, and written consent was obtained from all participants. West Midlands (Solihull) Research Ethics Committee (12/WM/0379) approved the study, Heart of England NHS Foundation Trust was the trial sponsor and the protocol was registered with Clinicaltrials.gov (NCT02994706).

Participants were screened from the West Midlands Adult CF Centre database by the study investigators. If eligible, the study investigators approached patients when they were attending routine clinic visits or during inpatient admissions to explain the study. If they were potentially interested, they were given the Participant Information Sheet. Having had time to consider the study and have any questions answered to their satisfaction, patients still keen to take part were asked to provide written informed consent. It was explained that they could withdraw from the study at any time.

At recruitment, baseline data were obtained and the participant was asked to take part in a semi-structured qualitative interview to assess the patient experience of their care to date and their expectations of participating in the study. Within strata, participants were then randomly assigned 1:1 to receive either HM or RC. The strata were based on the number of hospital admissions to receive intravenous antibiotics over the preceding 24 months, separated into four strata: ‘1–2 admissions’, ‘3–4 admissions’, ‘5–6 admissions’ and ‘more than 7 admissions’. The randomisation was completed by the selection of randomly ordered sealed opaque envelopes, with randomisation having been performed by a clerical member of the CF team with no involvement with the study.

### Interventions

#### RC cohort

These participants continued receiving routine CF care, including regular outpatient clinic appointments and inpatient admissions if required.

#### HM cohort

Participants randomised to receive HM were provided with a Bluetooth-enabled digital spirometer and a ‘Microsoft Windows’ enabled mobile phone and shown how to use the equipment before going home. Participants were asked to monitor their health status each Monday and Thursday during the 12-month study period. They were asked to record their FEV_1_ using a digital spirometer and record their symptoms using the Cystic Fibrosis Respiratory Symptom Diary – Chronic Respiratory Infection Symptom Score (CFRSD-CRISS) on their mobile phone.^5^ This is a validated disease-specific tool (consisting of 16 questions) designed to detect symptoms of PEx in CF, taking around 5 min to complete.

Data regarding lung function and symptoms were automatically transmitted to a specially designed website via a secure encrypted wireless connection, which the research team was able to access instantaneously. The mean values of the first 2 weeks’ data were used to determine the participants’ baseline levels for FEV_1_ and CFRSD-CRISS during the run-in phase. Following this initial 2-week period, the CF team received an alert if participants’ FEV_1_ or symptoms deteriorated below a set threshold. The criteria that triggered an alert were one of (1) a drop in FEV_1_ of 10% or more from baseline and (2) total score of the CFRSD-CRISS worsening by more than 10 points from baseline. Following an email alert, the research team attempted to contact the participant by telephone or text message within 24 h to discuss their symptoms and spirometry. Based on the outcome of this contact, the participant and the research team decided on the best approach. If the participant was felt to have a PEx requiring systemic antibiotics, a decision was made as to whether participants received a course of either oral or intravenous antibiotics depending on the severity of their symptoms, sputum microbiology results and patient choice. Participants in the HM group were provided with a prescription for a 14-day course of oral antibiotics at the baseline visit, with the choice of antibiotic based on their usual sputum microbiology. If participants received a course of antibiotics, we documented whether these were prescribed for a protocol-defined PEx (see definition below).

To optimise adherence to HM, the importance of adhering to the study protocol was reenforced at each study visit. The system also alerted the research team if any participant failed to record symptoms or spirometry data. If this occurred repeatedly, the research team contacted the participant to check if they were having any problems with the equipment and confirm whether they were willing to continue with the study. If there were any difficulties with using the equipment, these were resolved. If any participant failed to submit data for more than 20% of occasions over any 28-day period, we advised them that they risked being withdrawn from the study. If there was a good reason for them not to submit data (such as a foreign holiday of which we were not aware), we reminded them to inform us if this was the case. If the participant did not wish to proceed with the study at any stage, they were withdrawn and were reassured that this would have no adverse effect on their ongoing care.

In addition to recording their symptoms and spirometry, individuals receiving HM were asked to provide a urine sample once every week and send this urine sample in a prepaid envelope. These samples were batched and subsequently levels of biomarkers were analysed. The urinary biomarker data were not available to the research team during the course of this study. The results of this part of the study will be presented separately.

### Outcome measures

Before baseline, the number of courses of intravenous antibiotics and hospital admissions in the 2 years prior were recorded. We then aimed to record all quantitative outcome measures at baseline, 3-, 6-, 9- and 12-month study visits (Figure 1). Quantitative data collected at baseline included age, sex, body mass index (BMI; kg/m^2^), spirometry [FEV_1_, forced vital capacity (FVC)], concomitant medications and comorbidities.

#### Primary outcome measures

The coprimary outcome measures were the number of hospital inpatient bed days and HRQoL.

- **Hospital inpatient days** were defined by the number of complete 24-h periods between admission and discharge.
- **HRQoL** was measured by the Cystic Fibrosis Questionnaire – Revised (CFQ-R) Teen/Adult, a validated disease-specific HRQoL questionnaire,^19^ which consists of 49 self-reported items within 12 domains: (1) physical functioning (eight items); (2) vitality (four items); (3) emotional functioning (five items); (4) eating disturbances (three items); (5) treatment burden (three items); (6) general health perception (three items); (7) social functioning (six items); (8) body image (three items); (9) role limitations (four items); (10) weight problems (one item); (11) respiratory symptoms (six items); and (12) digestive symptoms (three items). Answers are reported on a 4-point scale rating frequency, difficulty, or truth and the scores range from 0 to 100, with a higher score indicating better quality of life.

#### Secondary outcome measures

- **Antibiotic requirements** were measured by total number of completed days on oral and intravenous antibiotics.
- **Protocol defined PEx** were recorded using the modified Fuchs criteria,^20^ which requires the presence of four or more of the following criteria:
  ● Change in sputum
  ● New or increased haemoptysis
  ● Increased cough
  ● Increased dyspnoea
  ● Malaise, fatigue or lethargy
  ● Temperature above 38°C
  ● Anorexia or weight loss
  ● Sinus pain or tenderness
  ● Change in sinus discharge
  ● Change in physical examination of the chest
  ● Decrease in pulmonary function by 10%
  ● Radiographic changes indicative of pulmonary infection
- **Spirometry (FEV_1_, FVC)** was performed using a digital Vitalograph spirometer in the CF outpatient clinic in accordance with the American Thoracic Society guidelines.^21^ The highest value for FEV_1_ and FVC obtained from three reproducible trials was recorded and compared with predicted normal values
- **BMI** (kg/m^2^) was measured using body weight (measured using digital scales) and height recorded using a wall-mounted tape measure
- **Anxiety and depression** were measured by the Hospital Anxiety and Depression Scale (HADS).^22^ This questionnaire is designed to detect and measure the severity of anxiety and depression. It consists of a series of 14 statements, with responses based on a 4-point Likert scale. The HADS is self-administered, with a higher score being indicative of greater anxiety or depression.

Cost-effectiveness analysis was conducted from an NHS and patient perspective. Participants were asked to record any costs related to their care throughout the study, including the cost of visiting the hospital for outpatient visits (such as travel and parking costs) and loss of pay due to time off work. Participants were asked to complete the EuroQol 5 dimensions (EQ-5D-5L), a generic HRQoL questionnaire^23^ and ICEpop CAPability measure for Adults (ICECAP-A), a generic measure of capability^24^ at baseline, 3, 6, 9 and 12-month study visits. The estimated costs associated with caring for each participant (such as costs of outpatient visits, inpatient admissions and staff time) and the cost of conducting the study (including staff costs and costs of the HM equipment) were recorded.

### Qualitative analysis

Semi-structured interviews were undertaken at baseline (all) and the 12-month study visit (HM cohort only). The aim was to explore factors that influenced participants’ participation, experiences and outcomes to support interpretation of the findings (e.g. differential uptake or use of HM, patient attrition).

Baseline questions focused on (1) reasons for participation, (2) disease experience including symptom-management, use of healthcare and impact of CF on quality of life and (3) expectations for HM. Follow-up interviews were undertaken with the intervention cohort to explore their experiences of using HM, including issues around acceptability and sustainability. Questions explored the technical aspects of HM (e.g. ease of use), behavioural issues (e.g. perceived costs and benefits, necessary skill sets) and educational issues (e.g. adequacy of information and training). Suggestions for further improvement were also sought. Where appropriate, follow-up interviews examined reasons for study withdrawal.

### Statistical and data analysis

The characteristics of participants in the study were described. Outcomes were summarised by group. Incidence rates and 95% confidence intervals were calculated for exacerbations, oral and intravenous (IV) antibiotic use and bed days.

Cost-effectiveness was assessed by calculating cost per reduction in hospital inpatient bed days and cost per quality-adjusted life year (QALYs) gained, and appropriate sensitivity analyses were conducted.

Interview transcripts were imported into NViVo and analysed using directed qualitative content analysis^25^ to interpret meaning from the interview data in relation to the topics of inquiry, while allowing themes to emerge inductively from the data. To optimise rigour, data were organised using the Framework^26^ method and analysed as part of team approach, with an audit trail kept throughout. The qualitative and quantitative findings were integrated using established methods^27^ as part of the final analysis to help interpret, understand and explain the trial findings.

### Safety

The Trial Management Group (TMG) was responsible for day-to-day running of the trial and met on a monthly basis to ensure that the study is running smoothly. The Trial Steering Committee (TSC) met every 3 months to ensure that the study is run ethically. The Data Monitoring and Ethics Committee (DMEC) met every 3 months and its responsibilities included ensuring that the trial is recruiting individuals to schedule and assessing any adverse events associated with HM. The DMEC could have stopped the trial early if felt necessary and reported to the TSC after every meeting. The TMG, TSC and DMEC functioned independently of the sponsor and funding bodies.

## Results

### Recruitment and follow-up

Eighty-eight patients were randomised, 44 to receive HM and 44 to receive RC. We were unable to meet the target of recruiting 100 patients due to a lack of patients meeting the inclusion and exclusion criteria interested in participating.

Baseline characteristics of participants are displayed in Table 1. Thirteen participants (14.7%) withdrew from the study: seven from the HM arm (16%) and six from the RC (14%) arm. The flow of participants through the study and reasons for withdrawal are displayed in Figure 1. Two participants died from progressive CF lung disease during the study, both in the RC arm.

**Table 1.** Baseline characteristics of participants.

Characteristic	Home monitoring group	Routine care group	All participants
*N*	44	44	88
Recruitment location; *n* (%)			
Inpatient	27 (61.4)	19 (43.2)	46 (52.3)
Outpatient	17 (38.6)	25 (56.8)	42 (47.7)
Sex; *n* (%)			
Female	16 (36.4)	26 (59.1)	42 (47.7)
Male	28 (63.6)	18 (40.9)	46 (52.3)
Age; mean (SD)	28.4 (8.8)	28.6 (10.1)	28.5 (9.5)
F508del homozygous; *n* (%)	25 (57)	23 (52)	24 (55)
BMI; mean (SD)	21.61 (4.6)	22.4 (4.8)	22.0 (4.7)
Chronic *Pseudomonas; n* (%)	42 (95)	41 (93)	83 (94)
FEV1 % predicted; median [Q1, Q3]	56.3 [47.0, 78.0]	59.5 [47.4, 80]	56.8 [47.1, 79.0]
*N*	42	42	84
Total IV courses in the last 24 months; *n* (%)			
1	15 (34.1)	11 (25.0)	26 (29.6)
2	5 (11.4)	8 (18.2)	13 (14.8)
3	3 (6.8)	4 (9.1)	7 (7.8)
4	4 (9.1)	1 (2.3)	5 (5.7)
5	2 (4.6)	3 (6.8)	5 (5.7)
6+	15 (34.1)	17 (38.6)	32 (36.4)
Median [Q1, Q3]	3 [1, 6.5]	3 [1, 6.5]	3 [1, 6.5]
Mean (SD)	4.1 (3.5)	4.5 (3.5)	4.3 (4.5)
Range (min, max)	(1, 17)	(1, 13)	(1, 17)
Home IV days in the last 24 months			
Median [Q1, Q3]	6.5 [0, 19]	8 [0, 43]	7 [0, 27.5]
Mean (SD)	16.3 (27.3)	26.3 (36.8)	21.2 (32.6)
Range (min, max)	(0, 152)	(0, 132)	(0, 152)
Hospital IV days in the last 24 months			
Median [Q1, Q3]	28 [11, 62.5]	21.5 [11.5, 60]	23.5 [11.5, 61]
Mean (SD)	40.0 (37.6)	40.8 (42.6)	40.3 (40.0)
Range (min, max)	(1, 167)	(5, 159)	(1, 167)
Total IV days in the last 24 months			
Median [Q1, Q3]	48.5 [14, 87.5]	55 [21.5, 96.5]	48.5 [18, 93]
Mean (SD)	56.0 (46.9)	67.1 (52.9)	61.5 (50.0)
Range (min, max)	(7, 234)	(9, 195)	(7, 234)
% IV days in hospital in the last 24 months			
Median [Q1, Q3]	90.9 [57, 100]	63.5 [28.5, 100]	83.3 [40.5, 100]
Mean (SD)	75.7 (30.8)	62.1 (35.0)	68.9 (33.5)
Range (min, max)	(4, 100)	(6, 100)	(4, 100)

BMI, body mass index; FEV_1_, forced expiratory volume in one second; IV, intravenous.

### HM adherence

Twenty-two (50%) participants in the HM group transmitted data at least once per week in more than 80% of their follow-up weeks. Adherence with twice-weekly data transmission was much lower, with only one (2%) participant being more than 80% adherent.

#### Primary endpoints

*Hospital inpatient days.* Mean number of days was 18.5 for the HM group (*n* = 39) and 18.6 for the RC group (*n* = 40) (Table 2).

**Table 2.** Antibiotic usage, exacerbations and hospital inpatient day incidence rate per year (95% CI) by group.

IR per year (95% CI)	Home monitoring group		Routine care group	
	Before^a^	After^b^	Before^a^	After^b^
IV courses	2.07 (1.78, 2.39)	–	2.25 (1.95, 2.59)	–
	*N* = 44		*N* = 44	
Home IV days	8.13 (7.54, 8.74)	–	13.16 (12.41, 13.94)	–
	*N* = 44		*N* = 44	
Hospital inpatient days	19.88 (18.95, 20.83)	–	20.38 (19.44, 21.34)	–
	*N* = 44		*N* = 44	
Total IV days	28.00 (26.91, 29.13)	30.00 (28.28, 31.74)	33.53 (32.33, 34.77)	26.93 (25.34, 28.58)
	*N* = 44	*N* = 39	*N* = 44	*N* = 40
Protocol-defined exacerbations	–	4.37 (3.73, 5.09)	–	3.83 (3.22, 4.53)
		*N* = 38		*N* = 36
Courses of oral antibiotics	–	3.23 (2.69, 3.85)	–	2.12 (1.70, 2.64)
		*N* = 39		*N* = 39
Oral antibiotics days	–	50.69 (48.5, 53.0)	–	31.43 (29.71, 33.21)
		*N* = 39		*N* = 40
Hospital inpatient days	–	18.54 (17.21, 19.94)	–	18.56 (17.24, 19.97)
		*N* = 39		*N* = 39

CI, confidence interval; IR, interquartile range.

a 24 months before the study.

b 12 months during the study.

#### HRQoL

Median respiratory symptom scale of the CFQ-R score, at 12-month follow-up, was increased for the HM group compared with the RC group, 66.7 (Q1, Q3: 55.6, 77.8) and 58.3 (33.3, 75.0), respectively (Table 3). Results for the other domains of the CFQ-R score can be seen in Table 3.

**Table 3.** Characteristics at baseline, 3, 6 and 12 months by group.

	Home monitoring group					Routine care group				
	Baseline	3 months	6 months	9 months	12 months	Baseline	3 months	6 months	9 months	12 months
BMI										
Mean (SD)	21.61 (4.6)	22.0 (3.8)	22.8 (3.9)	23.2 (3.6)	23 (3.8)	22.4 (4.8)	22.8 (3.6)	22.1 (5.3)	22.0 (4.0)	22.6 (3.8)
*N*	44	36	37	32	35	44	33	31	29	34
FEV1 % predicted										
Median [Q1, Q3]	56.3 [47, 78]	50 [43, 67]	50.5 [36, 77]	55 [41, 71]	55.5 [38.5, 75]	59.5 [47.4, 80]	57 [42, 71]	67.5 [61.5, 87]	63.5 [46, 78.5]	56 [44, 76]
*N*	42	33	34	30	32	42	29	28	24	27
CFQ-R										
Physical										
Median [Q1, Q3]	58.4 [33.3, 72.9]	56.3 [37.5, 75]	50 [37.5, 83.3]	70.8 [41.7, 83.3]	56.3 [29.2, 75]	45.8 [33.3, 87.5]	41.7 [16.7, 75]	47.9 [20.8, 87.5]	54.2 [29.2, 79.2]	48.5 [18.8, 91.7]
*N*	36	30	29	22	30	34	30	24	22	28
Vitality										
Median [Q1, Q3]	41.7 [33.3, 62.5]	50 [33.3, 58.3]	50 [33.3, 66.7]	50 [33.3, 66.7]	50 [41.7, 66.7]	37.5 [25, 58.3]	41.7 [33.3, 50]	41.7 [33.3, 56.9]	33. 3 [25, 50]	50 [25, 58.3]
*N*	36	30	29	22	30	34	30	24	22	28
Emotion										
Median [Q1, Q3]	70 [46.7, 86.7]	76.7 [46.7, 93.3]	80 [60, 100]	86.7 [66.7, 93.3]	66.7 [53.3, 93.3]	66.7 [53.3, 80]	66.8 [40, 83.3]	76.7 [56.7, 93.3]	70.0 [33.3, 86.7]	73.3 [53.3, 86.7]
*N*	36	30	29	22	30	34	30	24	22	28
Eating										
Median [Q1, Q3]	94.4 [66.7, 100]	94.5 [66.7, 100]	88.9 [66.7, 100]	88.9 [55.6, 100]	88.9 [77.7, 100]	100 [66.7, 100]	100 [66.7, 100]	88.9 [77.8, 100]	94.4 [66.7, 100]	94.4 [61.1, 100]
*N*	36	30	29	23	29	33	29	24	22	28
Treatment burden										
Median [Q1, Q3]	55.6 [38.9, 66.7]	55.6 [50, 66.7]	55.6 [44.4, 66.7]	55.6 [44.4, 77.8]	44.4 [55.6, 66.7]	55.6 [33.3, 66.7]	55.6 [44.4, 66.7]	55.6 [22.2, 66.7]	44.4 [33.3, 55.6]	44.4 [33.3, 55.6]
*N*	36	30	29	23	30	32	29	23	22	28
Health perception										
Median [Q1, Q3]	55.6 [44.4, 66.7]	55.6 [33.3, 66.7]	55.6 [44.4, 66.7]	55.6 [33.3, 66.7]	55.6 [44.4, 66.7]	55.6 [44.4, 77.8]	55.6 [22.2, 66.7]	55.6 [22.2, 66.7]	55.6 [33.3, 66.7]	44.4 [33.3, 66.7]
*N*	36	30	29	23	29	33	30	23	21	28
Social										
Median [Q1, Q3]	66.7 [47.2, 83.3]	63.9 [61.1, 77.8]	72.2 [55.6, 83.3]	72.2 [60, 83.3]	61.1 [55.6, 77.8]	66.7 [38.9, 77.8]	55.6 [38.9, 72.2]	72.2 [33.3, 83.3]	50 [38.9, 72.2]	61.1 [36.1, 75.6]
*N*	36	30	29	23	29	33	30	23	22	28
Body image										
Median [Q1, Q3]	77.8 [55.6, 88.9]	72.2 [33.3, 100]	66.7 [55.6, 83.3]	66.7 [44.4, 100]	77.8 [50, 100]	66.7 [55.6, 88.9]	66.7 [55.6, 88.9]	66.7 [44.4, 100]	66.7 [44.4, 100]	66.7 [44.4, 88.9]
*N*	36	30	29	23	29	33	29	23	22	28
Role										
Median [Q1, Q3]	70.9 [50, 83.3]	75 [58.3, 87.5]	83.3 [62.5, 100]	75 [56.9, 87.5]	75 [58.3, 100]	66.7 [50, 83.3]	66.7 [50, 75]	66.7 [45.9, 87.5]	66.7 [54.2, 91.7]	66.7, [45.8, 95.8]
*N*	36	28	28	24	29	31	29	24	20	28
Wt										
Median [Q1, Q3]	66.7 [33.3, 100]	66.7 [33.3, 100]	66.7 [33.3, 100]	66.7 [33.3, 100]	66.7 [33.3, 100]	100 [50, 100]	100 [33.3, 100]	100 [33.3, 100]	100 [66.7, 100]	100 [33.3, 100]
*N*	36	29	29	24	29	32	29	24	22	28
Resp										
Median [Q1, Q3]	61.1 [44.4, 75.0]	61.1 [50, 66.7]	66.7 [50.0, 75]	66.7 [47.2, 80.6]	66.7 [55.6, 77.8]	63.9 [44.4, 77.8]	55.6 [38.9, 72.7]	61.1 [33.3, 72.2]	55.6 [38.9, 69.4]	58.3 [33.3, 75]
*N*	36	30	28	24	29	32	27	23	20	28
Digest										
Median [Q1, Q3]	88.9 [77.8, 100]	94.5 [77.8, 100]	88.9 [77.8, 100]	77.8 [72.2, 94.4]	88.9 [66.7, 100]	88.9 [72.2, 100]	77.8 [55.6, 100]	88.9 [66.7, 100]	77.8 [66.7, 88.9]	88.9 [66.7, 100]
*N*	36	30	28	24	29	32	27	23	21	28
HADS										
*N*	33	24	28	21	30	34	28	22	21	26
Anxiety										
Median [Q1, Q3]	6 [4, 10]	6 [3.5, 10.5]	5 [2.5, 9.5]	4 [2, 6]	6 [3, 11]	5.5 [3, 10]	6.5 [4, 12]	5 [2, 7]	7 [4, 10]	5 [4, 10]
Depression										
Median [Q1, Q3]	3 [1, 6]	3 [1.5, 6.5]	4 [1, 6]	3 [1, 7]	5 [1, 8]	3 [2, 7]	4.5 [1, 7]	2 [1, 5]	4 [1, 8]	3 [1, 8]

BMI, body mass index; CFQ-R, Cystic Fibrosis Questionnaire – Revised; FEV_1_, forced expiratory volume in one second; HADS, Hospital Anxiety and Depression Scale; SD, standard deviation.

#### Secondary endpoints

In the HM group, 4.4 (3.7–5.1) protocol-defined PEx were detected during the study period compared with 3.8 (3.2–4.5) in the RC group. The HM group received 30.0 (28.3–31.7) days on IV antibiotics compared with 26.9 (25.3–28.6) in the RC group. Participants in the HM group received 50.7 (48.5–53.0) days of oral antibiotics for protocol-defined PEx, compared with 31.4 (29.7–33.2) days in the RC group. Results for the other secondary outcomes can be seen in Table 2.

### Cost-effectiveness analysis

The total mean bootstrapped NHS costs with 95% CI were approximately £1500 more per patient for the RC arm than the HM group, although this was not statistically significant (*p* = 0.792). This is also reflected in the wider 95% CI and this result is consistent with the longer inpatient stays associated with the RC arm. Patient costs, which included travel costs, over-the-counter medications and any loss of pay, were slightly higher (difference £124, *p* = 0.293) for the RC group than the HM group. Taking all this into account, the total societal (NHS and patient) costs were approximately £1650 more per patient for the RC group than the HM group, a difference although again this was not statistically significant (*p* = 0.775). Due to the complexity, the full cost-effectiveness methods and analysis will be presented in a separate manuscript.

### Qualitative analysis

Qualitative analysis provided in-depth understanding about the factors that shaped the experience and impact of HM. These will be explored at length in a separate paper, but are summarised here to contextualise the main results and inform future research.

#### Motivations for participation in the trial

Despite having no previous experience of HM, most participants had a positive stance towards HM, expecting that it would confer personal benefits to those in the intervention group. They theorised how HM might lead to better heath by giving patients more ‘control’ over their condition, rather than feeling like they were ‘sitting in the back seat’. This was a key motivation for participation. In particular, participants felt that HM would help them to detect subtle changes in their condition, enabling more responsive and preventive approaches to care. For example, they described how HM might support them to ‘pick up infection[s] before they became too problematic’ and ‘plan everything else a bit better’. Participants hoped this would ‘cut down on clinic visits’ and reduce hospital admissions.

Participants also envisaged that regular test results would support better understanding of their condition and treatment, as suggested by this individual who stated, ‘mentally it would be better for me … because I can see that’s formed a bit of a pattern’. It was felt that this information would promote more positive health behaviours. It was expected that poor results could ‘push’ participants ‘to act’ by increasing treatments or accessing earlier help from the multidisciplinary team (MDT). In contrast, improved results could reinforce adherence by helping patients to ‘know I’m not doing my treatment for nothing’.

#### Anticipated challenges

Participants raised few concerns about the trial or HM. However, when challenges were discussed, these generally related to the frequency of producing HM results. In part, this was a practical issue. Participants acknowledged that ‘remembering to do it’ and ‘finding the time’ might be difficult, given that ‘some people already feel overwhelmed by what they’ve got to manage’. However, there was also concern that frequent monitoring may become ‘a stressful thing’ or cause them to become ‘too obsessive’. Protocol adherence was also raised, with some participants wondering if self-protective behaviours might cause them to avoid using HM, if they could ‘guess that the blows are going to be bad’.

#### Reasons for withdrawal

Reasons for withdrawal were generally linked to the evaluation, with participants feeling unable to complete the battery of questionnaires and/or provide weekly urine samples. However, withdrawal was occasionally linked to the emotional consequences of HM which was reported to increase anxiety levels in some participants by (1) requiring them to deal with their CF more frequently than usual or (2) presenting them with disappointing spirometry results. For these individuals, the negative aspects of HM were perceived to outweigh the potential benefits, imposing data on them – that they felt unable to manage.

#### Experiences of the HM

Participants’ baseline expectations were largely met, with most describing HM as a beneficial intervention. A recurrent theme was the positive impact of HM on their awareness of their own symptoms, with participants reporting that regular home spirometry results ‘educated me’, ‘opened my eyes up’ and made them ‘more aware of what’s going on in your own body’. They also described how it supported them to develop a better understanding of the complex relationship between their symptoms, treatments and other influencing factors on their health. It gave them ‘an idea of where I am . . . and what kind of treatment is working for me’ and better ‘understand why it’s going low and why it’s going high’.

HM was also described as an intervention that ‘promotes independence’ and helped participants to ‘keep in control’. They described how HM meant that you were ‘not reliant on other people to tell you that you’re ill’ but allowed you to ‘see it for yourself’. For many, having an increase sense of self-agency was felt to prompt better self-care and help-seeking behaviours, as described by this participant: ‘it just made me a bit more aware, maybe that I was, getting an infection and that maybe I do need to reach out to get care’. For some, it also supported a greater sense of urgency to ‘do something about it straight away’.

HM was also felt to ‘motivate’ people to adhere to their treatments, by providing a visible means to see their impact: ‘I think the main thing is about concentrating on my respiratory treatments because these are the main things that will affect my blows’. On one hand, HM could provide ‘reassurance’ or ‘piece of mind’ that treatment was working by showing lung function was stable, despite feeling symptomatic. On the other hand, evidence of deteriorating lung function could make participants ‘more aware of what’s going on & probably try a little bit harder’ or feel ‘more determined’. Thus, for many participants, HM provided ‘another tool’ and ‘another piece of the jigsaw’ to positively inform self-care decisions.

With regard to the psychological impact of HM, most found it had a positive influence on their mental health, helping them to ‘worry less’ and feel ‘more relaxed because I’ve been able to look for signals of an infection so I’ve been more confident that I’m going to get over it quicker and I’ll have more time to do normal things’. Participants also felt that it gave them confidence to acknowledge and respond to changes in their condition, ‘rather than just burying it’. Some spoke about the isolation of living with CF and how the additional contact with the clinical team during deteriorations was comforting; ‘it’s like they are picking you up and holding you up’ or that they ‘would sort of explain it and … help me think about it in a better way’. This made them feel ‘very close to the hospital’ and ‘easier to describe and express my feelings about Cystic Fibrosis’.

A minority, however, found the HM more challenging, explaining how frequent monitoring caused them to be ‘a bit more anxious or a bit more worried’, frustrated or annoyed, especially when results deteriorated. Reflecting the reasons given in the withdrawal interviews, a small number of participants described how they had become a ‘bit more paranoid’ or how spirometry had become ‘like an obsession that you want to know all the time’.

#### Suggestions for the future

Recommendations for the future HM included more flexibility and choice. Participants wanted more say over the frequency of monitoring, with once a week being a preferred option, and more choice about when spirometry is performed (based on their existing commitments and extending the monitoring window to beyond 11 pm).

### Adverse events

The proportion of participants reporting serious adverse event (SAE’s) was similar between groups and all SAEs were deemed unrelated to the study intervention.

## Discussion

Several investigators have reported the use of telehealth or HM in people with CF, but the vast majority have been small feasibility studies with limited external validity.^6–15^ Lechtzin *et al.*^17^ recently reported the results of the eICE study, the first large multicentre randomised controlled study of HM in CF. The primary outcome (absolute change in FEV1 over the 52-week study period) was negative, with no evidence of slowing lung function decline over the 12-month study period in the HM group. However, HM was found to be feasible and able to detect more PEx than RC, with PEx detected by HM being more likely to be treated with oral antibiotics and less likely to require intravenous antibiotics. Importantly, there were no cost-effectiveness analyses included in the Lechtzin study, no assessment of anxiety or depression and no qualitative assessment of the patient experience.

The results of this study are in agreement with those of Lechtzin *et al.*, with more PEx being detected in the HM group and more of these PEx being treated with oral antibiotics. We saw no difference in inpatient bed days or HRQoL. Similar to Lechtzin *et al.*, we found no difference in FEV_1_ or BMI over the 12-month study period. This study does however add a rich qualitative assessment of the patient experience as well as a cost-effectiveness analysis from a patient and healthcare provider perspective. Participants using HM provided generally very positive feedback, feeling supported by the intervention and reassured that their health was being assessed between clinic visits. Participants felt that HM gave them the confidence to make independent decisions about their health and prompted appropriate help-seeking, as well as enabling them to take control and be proactive at self-managing changes in their condition. The cost-effectiveness assessment demonstrated a trend towards lower cost of providing HM compared with routine CF care, even when factoring in the cost of equipment and staffing to respond to the results.

In this study, use of oral antibiotics was facilitated by the study design, in which participants in the HM cohort were provided with a prescription for a 14-day course of oral antibiotics to be kept at home in case they developed a PEx. There is a potential that the increased use of oral antibiotics could cause unintended consequences, such as increased bacterial resistance and adverse effects such as *Clostridium difficile* infection and antibiotic allergies. There was no evidence of increased adverse events to support this suggestion over a 12-month study period, but if HM was to be introduced into clinical practice, this would need to be assessed over a longer time frame. In addition to these overt adverse effects, more subtle effects on the airway and gut microbiome would need to be considered.

We acknowledge several limitations that should be borne in mind when interpreting the results of this study. This was a small single-centre pilot study and lack of patients eligible and willing to participate resulted in our failure to recruit the target of 100 participants. Despite this, our cohort represented a diverse group of patients, with the HM group including adults with a wide age range (18–55 years), severity of lung function (27–105% predicted FEV_1_) and social context, which suggests that HM may be an acceptable approach to a wide range of adults with CF. Limiting the study to one centre reduced costs and meant that the research team was familiar with all participants and could act upon alerts using that knowledge, but low patient numbers make the results less reliable. Adherence to the HM intervention was lower than we hoped, although 50% of individuals transmitted data at least once per week in more than 80% of their follow-up weeks, which is comparable with adherence observed by Lechtzin *et al.*^17^ However, adherence with twice-weekly data transmission was lower in this study, with only 2% of participants being more than 80% adherent, which makes the effects of the intervention harder to assess. Before conducting this study, we assessed the accuracy of the handheld spirometer device compared with results from our clinic spirometer device and found the results were on average 2% lower, although results were reproducible (data not presented). This observation has recently been replicated in a study by a separate group^28^ and although this means results from the two devices cannot be directly compared, we are satisfied that the handheld spirometer device provides adequate results for the purposes of HM.

The 12-month follow-up interviews suggest that withdrawal was generally linked to the evaluation burden, rather than the intervention. Given that participants anticipated few problems with participation at baseline, the findings highlight the important role of managing expectations about research involvement. Retention may also be improved by identifying a minimum set of outcomes that should be measured in future clinical trials of HM. It may also be useful to examine how the frequency of lung functioning monitoring could be reduced to respond to patient’s suggestions to make it more manageable – without losing clinical benefit. Indeed, the qualitative data suggest that HM may exacerbate health-related anxiety or frustrations for some individuals. It would therefore be important to examine how HM can be tailored to patient preferences, with support for well-being before, and during use.

Due to the nature of the intervention, we were obviously unable to blind participants to allocation to study groups. Where feasible, we ensured that the clinical team making treatment decisions were not influenced by allocation to study groups, but we cannot exclude the possibility that this may have influenced patient care. None of the individuals in this study were taking cystic fibrosis transmembrane conductance regulator (CFTR) modulators, as these medications were not widely available at the time of recruitment and this limits the applicability of the findings. The HM equipment in general functioned well, although in some cases, spirometers or mobile phones needed to be replaced. Towards the end of the study, the company that developed the bespoke website (Safe Patient Systems Ltd.) went into liquidation, resulting in participants in the HM group prematurely stopping the HM intervention. This only affected four participants for the last few weeks of their participation and we do not feel that this affected the overall results but this highlights the potential problems inherent in relying on a third party for technology support.

The improving survival of people with CF is resulting in increasing numbers of people born with this condition living well into adulthood. Although overwhelmingly positive, this growth in numbers of adults with CF makes the feasibility of providing the current standard model of regular face-to-face CF clinical encounters more challenging. Constraints on healthcare budgets worldwide make the provision of additional resources, including additional staff and new CF centres, less realistic. Novel means of using technology to improve the interaction between people with CF and their MDT provides a potential solution to this increasingly difficult conundrum.^29^ HM potentially reduces the requirement for hospital visits and therefore could reduce opportunities for cross-infection, although this must be balanced against the theoretical benefits of regular direct patient contact with the MDT.

Importantly, the qualitative results of this study demonstrate that most participants welcomed HM as an intervention for CF and were able to integrate it into their daily lives. HM was considered beneficial to health, increasing self-awareness of their condition and prompting a range of self-care strategies. It was also described as a ‘supportive’ intervention that could facilitate access to healthcare in a responsive manner, rather than a replacement for patient–staff contact. HM also appeared to be user-friendly with few people reporting technical barriers. Indeed, HM was acceptable to diverse patients across a wide range of age groups, technical abilities and preexisting treatment burdens. However, that is not to say that all participants responded the same way. Those who demonstrated a baseline eagerness to learn more about their condition appeared more engaged with the intervention during the study and potentially gained the most benefit. A minority, however, found HM more challenging to sustain, suggesting that some patients will need additional support to gain optimal benefit, including those with health-related anxiety and lower self-agency. The full potential of HM may therefore require further work to make it more patient-centred, including increased choice, flexibility and support.
